# Balance Disorders: Insufficient Supply of Vestibular Examinations by the Italian National Health Service, 2021–2023

**DOI:** 10.3390/healthcare14111544

**Published:** 2026-06-01

**Authors:** Luciano Bubbico, Giuseppe Mastrangelo, Fabio Barbone, Luca Cegolon

**Affiliations:** 1 Hearing Loss Research Group, Department on Sensorineural Disability, National Institute of Public Policy (INAPP), 00198 Rome, Italy; 2 University of Padua, 35218 Padua, Italy; 3 Inter-Departmental Center for Medical Sciences (CISMED), University of Trento, 38100 Trento, Italy; 4 Department of Medical, Surgical & Health Sciences, University of Trieste, 34129 Trieste, Italy; 5 Public Health Unit, University Health Agency Giuliano Isontina (ASUGI), 34129 Trieste, Italy

**Keywords:** vestibular function, presbiastasis, balance disorders, nystagmus, audiology, access to care, telemedicine

## Abstract

**Background:** Vestibular tests are critical for an early detection of balance disorders, thereby reducing the risk of falls, particularly in older adults. The present is an ecologic study where the units of observation are the Italian Regions. Regional scores of access to essential levels of care (LEAs) were employed as predictors to investigate the hypothesis that vestibular examinations supplied by the Italian National Health Service (NHS) were insufficient to meet the needs of the Italian population. **Methods:** The number of first-level (clinical evaluations of vestibular function) and second-level (recorded spontaneous nystagmus, induced nystagmus, or rotary stimulation) vestibular tests per 100 K population was estimated by Italian region and calendar year during the 2021–2023 period. The odds (i.e., number of any second-level vestibular tests divided by difference between number of first- and second-level tests) by region and calendar year were investigated as a proxy for regional propensity to refer patients to a second-level test following a first-level vestibular assessment. A logistic regression investigated the odds by region and calendar year. Lastly, the regional number × 100 K population (prevalence) of vestibular examinations underwent linear regression analysis, using LEAs as predictors. **Results:** Descriptive analysis showed that first-level assessments were the most common vestibular tests in Italy during the 2021–2023 period. Prevalence of first-level vestibular examinations was not associated with any indicator of access to healthcare in linear regression. By contrast, the prevalence of second-level vestibular tests decreased with social inequality yet increased with the indicator of higher access to hospital care. In logistic regression, referral propensity to second-level vestibular tests progressively decreased from 2021 to 2023 and exhibited considerable regional variability, being lower than in Lombardy (reference) in all other Italian regions but Veneto (aOR = 4.826; 95%CI: 4.445; 5.329) and Trento autonomous province (aOR = 1.488; 1.363; 1.624). **Conclusions:** The number of vestibular function tests supplied by the National Health Service in Italy during the 2021–2023 period was probably insufficient to meet the care needs of the general population, forcing more than 66.8% of patients to forgo vestibular evaluation or turn to private audiological services with out-of-pocket payments. The shortfall was greater for more costly instrumental (second-level) vestibular tests, whose supply was influenced by social inequalities and barriers to accessing audiology care at the regional level. The National Recovery and Resiliency Plan has allocated EUR 20.23 billion for healthcare services in Italy, with the aim of addressing patient care requirements in every area of the country.

## 1. Introduction

Symptoms of vertigo, dizziness and disequilibrium are common complaints among patients of all ages, especially those aged > 60. Balance disorders can have vestibular or extra-vestibular origins. Dizziness of vestibular origin affects 15% of patients > 65–70 years old, whereas a third of patients older than 60 and half of individuals older than 85 reportedly exhibit balance disorders of non-vestibular origin [1,2,3].

The vestibular system is made up of specialized structures—semicircular canals, known as the saccule and the utricle, and otoliths—in the inner ear, which play a significant role in maintaining balance and spatial orientation by detecting changes in head position and movement, thereby sustaining the upright and stable stance of the human body. The vestibular system also contributes to eye movement control and spatial awareness.

The vestibular function is anatomically and physiologically linked with the hearing function inside the internal ear and deteriorates with age [4]. Increasing life expectancy requires additional efforts to meet the growing needs of an aging population, especially in high-income countries. Deterioration of the vestibular system (presbiastasis) is a frequent condition in advanced age, causing walking difficulties and increasing the risk of falls, thereby compromising everyday activities, social interactions, and self-esteem and contributing to social isolation, eventually leading to cognitive decline [5,6].

The vestibular examination combines both clinical (first-level or bedside assessment) and instrumental tests (second-level assessments). First-level assessment includes an accurate medical history taking—type of symptoms, duration of episodes, triggers (head position, movements, and stress), and associated symptoms (nausea, vomiting, and hearing loss)—a clinical examination (evaluating spontaneous nystagmus and static/dynamic balance), and physical maneuvers to assess the patient’s response to stimuli, such as head and eye movements and the vestibulo-ocular reflex (VOR) [7]. The latter procedures allow for a diagnosis in most patients with vestibular disorders [7,8,9,10,11]. In a subgroup of patients, vestibular function testing may be invaluable in reaching a correct diagnosis and, ultimately, in the optimal treatment of these patients. Second-level examinations may, therefore, be needed, including three objective diagnostic tests [12]:

- The mono-thermal and bi-thermal caloric test, involving water irrigation of the external auditory canal, a traditional test providing information on the vestibular performance by comparing the function of horizontal canals on both sides [13,14].
- The rotary stimulation test, which uses a computer-controlled rotary chair with low-frequency sinusoidal rotations [15].
- The spontaneous nystagmus test studies eye movements, recording the chorio-retinal potential using surface electrodes (electrooculography, EOG) or through infrared video-oculography (VOG).

Recently, the video Head Impulse Test (vHIT) and the functional Head Impulse Test (fHIT) have become available to record the vestibulo-ocular reflex (VOR) [16,17]. Finally, it is now possible to study the functionality of the saccule and the lower portion of the vestibular nerve up to the central nervous system through cervical vestibular-evoked myogenic potentials (cVEMPs) [18].

The admixture of distressed, at-risk patients, diagnostic uncertainty, and time pressure has led to an increase in the number of vestibular tests performed [19,20,21], which consistently rank among the most billed office procedures associated with audiology practices in the United States [22] and are also frequently performed by other specialists and primary care clinicians [23]. However, there is no “standard practice”, and the use of vestibular tests varies markedly among geographic regions, medical specialties, and practice patterns [24,25,26].

The vestibular system remains mysterious, despite more than 150 years of research. Although several studies have reported on tests of the vestibular system as a valid and reliable, evidence-based screening battery for easy clinical use, there is no agreement on which method is optimal depending on the clinical case, and clinical choices are often made on an individual, regional, institutional bases and in the absence of standardized guidelines and consensus [1]. In brief, there is no efficient use of vestibular testing [12].

We investigated Google Scholar to detect articles on the use of vestibular tests in, separately, “ecologic studies” or “geographic studies”. The web search engine returned 31 and 21 reports, respectively, but none was found on the insufficient supply of vestibular examinations by healthcare services. Despite the significant number of publications on vestibular function testing, geographic studies are uncommon due to a lack of large cohorts available [26].

### 1.1. Overview of the Italian National Health Service

The Italian National Health Service (NHS) is a universal system funded by central taxation and centered around primary care physicians, who are responsible for referring patients to a higher level of care [27]. Whilst primary care services are predominantly free of charge, access to planned (i.e., not emergency) specialized healthcare and medicines is subject to a subsidy, unless patients are exempt for low income or a recognized chronic health condition linked to the referral to a higher level of care or prescribed drug.

The delivery of healthcare is decentralized, and each Italian region is responsible for the organization and supply of health services through the respective local health units, which in turn oversee the delivery of healthcare within their territorial catchment area. The 20 Italian regions (Trentino Alto Adige region is broken down by the autonomous provinces (AP) of Trento and Bolzano) function as public entities with political, administrative and organizational autonomy. Nonetheless, the Italian Government supervises the supply of the essential levels of healthcare (LEAs), including audiology services, to all Italian citizens under conditions of universality, equity and appropriateness. Healthcare facilities funded by the Italian NHS include public institutions (whose staff and managers are employed or hired by local health units) and publicly funded private centers, which have staff and organization independent from the local health units [28].

### 1.2. Aims

Our aim is to appraise the nationwide supply of vestibular examinations (primary assessment of the vestibular function and instrumental tests) across the Italian regions and calendar year during 2021–2023. Since ecologic studies have limited capacity to characterize exposure data, indicators of access to LEA were employed as predictors of regional differences. The hypothesis was that vestibular examinations supplied by the Italian NHS are insufficient to meet the audiological care needs of the general population.

## 2. Methods

### 2.1. Ethical Considerations

The present study was approved by the ethical committee of the University of Trieste (Prot. N.1; 27 January 2026) and was conducted in compliance with the declaration of Strengthening the Reporting of Observational Studies in Epidemiology (STROBE).

### 2.2. Study Design

The present study is an ecologic study where the units of observation are the Italian Regions.

### 2.3. Data Sources

Anonymized data on vestibular examinations were retrieved from Agenas (National Agency for Regional Health Services). Individual records contained information on the region where tests were performed, calendar years (2021, 2022 and 2023), first-level exam (vestibular clinical examination), second-level vestibular tests (recorded spontaneous nystagmus, vestibular caloric test, vestibular rotary tests), and facility (examinations supplied by public audiology facilities (PAF) versus publicly funded private audiology facilities, PFPAF). Information on age, sex, residence, and final diagnosis was not available.

The number of consultant medical audiologists registered in each region in calendar year 2025 was retrieved from the National Federation of Provincial Medical Councils (FNOMCeO). The number of audiology health technicians registered in each region in 2025 was retrieved from the National Federation of the Council of Healthcare Professionals (FNOTSRM-PSTRP).

The number of residents in each region by calendar year was drawn from the Office for National Statistics of Italy (ISTAT) [29].

The regional levels of LEAs are monitored annually through numerical scores based on a set of approximately 100 relevant indicators. Using as source a publication of the Italian Ministry of Health [30], regional indicators of access to LEA in 2022, the last available year of observation and the intermediate year between 2021 and 2023 (the timeline of the current study), were retrieved for the following healthcare domains:

- **Prevention care**, the key sector for public and occupational health, concerned with protecting the health and safety of the general population and workers through health promotion, disease prevention, environmental risk control, and the promotion of healthy lifestyles. The indicator ranges from 0 to 100, with a benchmark ≥ 60 reflecting sufficient proportion of the general population accessing prevention services funded by the NHS. Increase in this indicator reflects higher access to preventative service funded by the Italian NHS.
- **District healthcare**, which refers to primary care, emergency care, pharmaceutical care, specialist outpatient care, home care (for the elderly, chronically ill, and non-autonomous patients), prosthetics, residential and semi-residential social care. The indicator ranges from 0 to 100, with 60 being the benchmark for sufficient proportion of the general population accessing district healthcare services funded by the NHS. Increase in this indicator reflects higher access to primary care services funded by the Italian NHS.
- **Hospital care**, the set of facilities, services, and professionals providing diagnosis, treatment, and rehabilitation of patients within a hospital. These facilities are managed independently or within larger Local Health Authorities. The indicator ranges from 0 to 100, again with 60 being the benchmark reflecting sufficient proportion of the general population accessing hospital services funded by the NHS. Increase in this indicator reflects higher access to hospital care services funded by the Italian NHS.
- **Social inequality**, an indicator measuring the share of the population forgoing access to NHS services due to barriers influenced by social inequalities. This indicator ranges from 0 to 100, and its increase reflects a higher proportion of the population giving up access to healthcare services funded by the Italian NHS.

The first three above indicators (ranging 0–100%) increased with the percentage of the regional population accessing LEA in any of the above three healthcare domains.

The fourth indicator (ranging 3–9%) reflected the proportion of the general population relinquishing healthcare due to barriers to health service access, particularly for situations of socio-economic disadvantage.

### 2.4. Statistical Analysis

The number of vestibular examinations per 100 K population was estimated in each region by calendar year. The frequency distribution and prevalence × 100 K population of consultant medical audiologists and health technicians was estimated in each region for calendar year 2025. For all prevalence × 100 K, the Italian census population as of 31 December of each year (2021, 2022, 2023) was used as denominator.

Multiple linear regression was fitted separately for prevalence per 100 K population of first- and second-level vestibular tests, including 8 linear terms as predictors:

- The four abovementioned LEA indicators for 2022 (social inequality, access to district healthcare, access to hospital care, access to prevention care);
- Prevalence of consultant medical audiologists and technicians per 100 K population registered in Italy in 2025.

Three multiple linear regression models were built up for each of the three study years, broken down by type of facilities (total facilities funded by the Italian NHS vs. PAF). Variables retained in the model were backward stepwise selected. Results were expressed as adjusted regression coefficients (aRC) with 95% confidence intervals (95%CI).

Assuming any second-level vestibular examination was performed following a first-level assessment, a referral propensity binary proxy indicator was generated, taking value of 1 for each second-level vestibular test performed by region and calendar year and 0 for all records corresponding to the difference between number of 1st- and 2nd-level vestibular tests by region and study year.

A multivariable logistic regression model was then fitted on the above binary referral propensity indicator, considering Lombardy as reference since it is the largest Italian region, having a population of approximately 10 million residents as of 31 December 2023. Results were expressed as adjusted odds ratios (aOR) with 95% confidence intervals (95%CI) of the regional propensity of referring patients to a second-level vestibular test after a first-level assessment, controlling for region and calendar year.

Statistical analysis was performed using Stata 14.3 (Stata Corporation, College Station, 254 TX, USA).

## 3. Results

**Figure 1:**
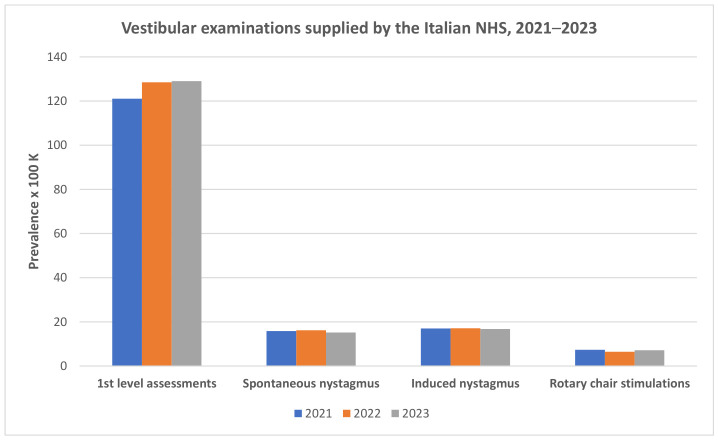
First-level clinical evaluation and second-level instrumental vestibular tests. Nationwide prevalence × 100 K population by calendar year 2021–2023). NHS = National Health Service.

**Figure 2:**
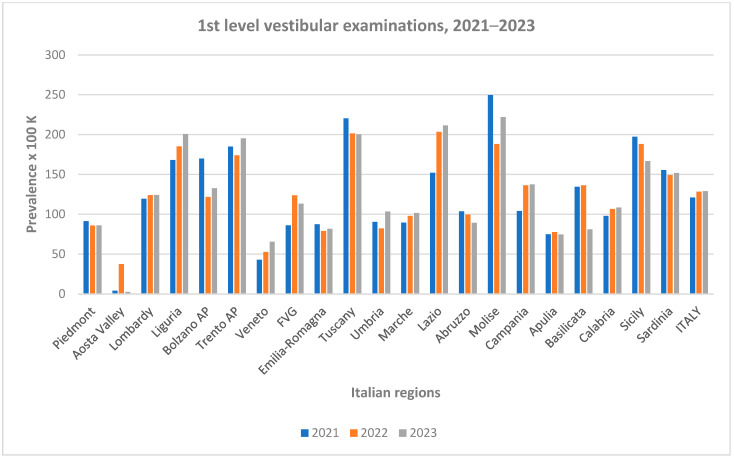
First-level vestibular examinations supplied by the Italian National Health Service (NHS), 2021–2023: prevalence × 100 K population by region and calendar year. FVG = Friuli Venezia Giulia; AP = Autonomous Province.

**Figure 3:**
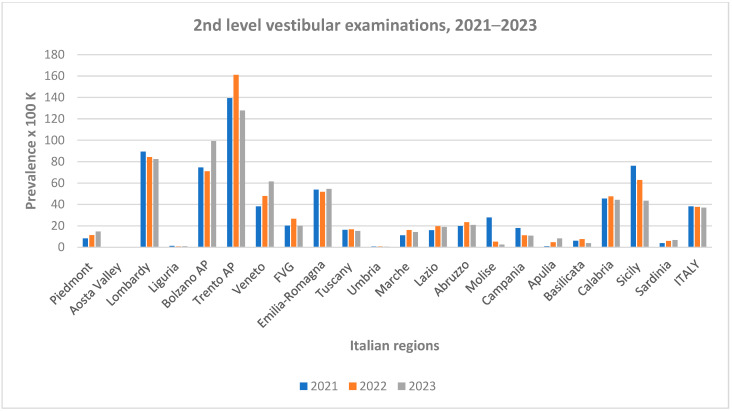
Second-level vestibular examinations supplied by the Italian National Health Service (NHS), 2021–2023: prevalence × 100 K population by region and calendar year. FVG = Friuli Venezia Giulia; AP = Autonomous Province.

**Figure 4:**
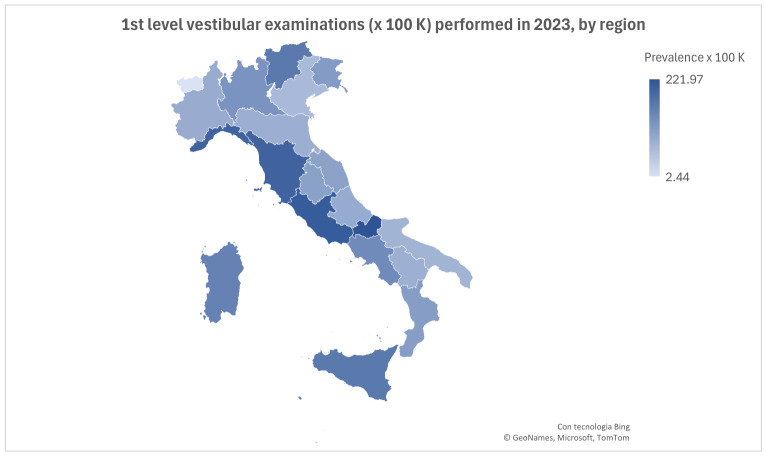
Prevalence × 100 K population of first-level vestibular examination supplied by the Italian National Health Service in 2023, by region.

**Figure 5:**
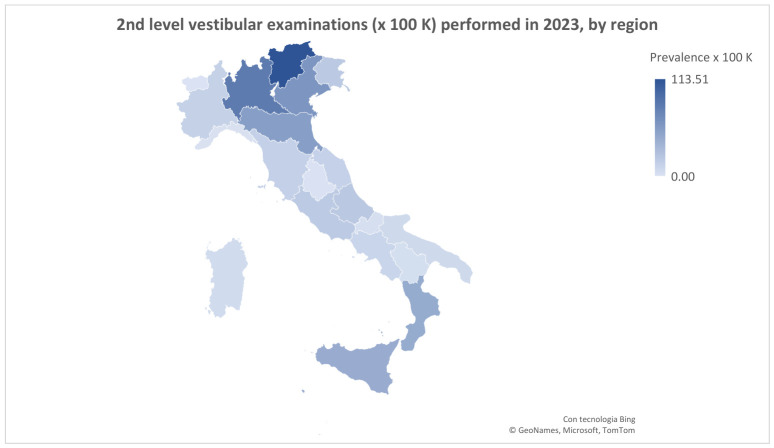
Prevalence × 100 K population of second-level vestibular examinations supplied by the Italian National Health Service in 2023, by region.

First-level examinations were by far the most common tests to assess the vestibular function in Italy during 2021–2023 (Figure 1, Figure 2 and Figure 3). Moreover, considerable regional variability for first- and second-level tests was observed (Figure 1, Figure 2, Figure 3, Figure 4 and Figure 5). Supply of both first- and second-level vestibular examinations was more prevalent in PAF rather than PFPAF across all regions and study years (Table 1, Table 2 and Table 3).

The nationwide prevalence × 100 K population of vestibular examinations slightly increased over time (from 121.08 in 2021 to 129.02 in 2023 for first level, whereas it slightly decreased from 38.24 × 100 K in 2021 to 37.02 × 100 K in 2023 for second-level vestibular examinations). Nationwide prevalence × 100 K population of first-level examinations increased by calendar year in PAF (from 92.57 in 2021 to 103.65 in 2023), whereas it slightly decreased in PFPAF (28.40 in 2021 vs. 27.93 in 2022 vs. 25.26 in 2023). Whilst increasing over time in PAF (from 21.19 in 2021 to 23.82 in 2022 and 24.31 in 2023), the nationwide prevalence × 100 K population of second-level vestibular examinations was slightly reduced in PFPAF (from 17.06 in 2021 to 13.80 in 2022 and 12.72 in 2023) (Table 1, Table 2 and Table 3).

The regional prevalence of vestibular tests × 100 K population was considerably uneven. Excluding data from small regions like Aosta Valley, Umbria and Molise, min/max values for first-level vestibular examinations (prevalence × 100 K population) were as follows: 42.93 (Veneto) vs. 220.41 (Tuscany) in 2021; 52.74 (Veneto) vs. 203.64 (Lazio) in 2022; 65.62 (Veneto) vs. 211.54 (Lazio) in 2023. The overall prevalence of second-level vestibular examinations × 100 K population ranged from 0.92 (Apulia) to 139.23 (Trento AP) in 2021, from 0.73 (Liguria) to 160.01 (Trento AP) in 2022 and from 0.93 (Liguria) to 127.67 (Trento AP) in 2023 (Table 1, Table 2 and Table 3).

Prevalence of spontaneous and induced nystagmus tests was considerably higher in Bolzano and Trento AP, followed by Emilia Romagna, Calabria and Sicily (Supplementary Table S1), whereas prevalence of rotary stimulations was higher in Lombardy, with a balanced distribution between PAF and PFPAF (lower prevalence of tests in PAF in 2021) and Veneto (where tests supplied by PAF prevailed) (Supplementary Table S1). In all 3 study years, Umbria and Aosta Valley did not perform any second-level tests, and Friuli Venezia Giulia (FVG) region did not deliver any induced nystagmus or rotary stimulation tests (Supplementary Table S1). Rotary stimulation tests were largely neglected, with the highest prevalence observed in Lombardy (increasing from 8.88 × 100 K in 2021 to 10.92 × 100 K in 2022 and 11.40 × 100 K in 2023) and Veneto (increasing from 5.24 × 100 K in 2021 to 9.98 × 100 K in 2022 and 14.06 × 100 K in 2023). In Bolzano AP, FVG and Aosta Valley regions, no rotary stimulation tests were executed in all three study years, both in PAF and PFPAF. In Basilicata, only one rotary stimulation test was performed in PFPAF in 2023 (Supplementary Table S1).

Table 4 displays the regional distribution of the referral propensity indicator in 2022, ranging from 0.004 (Liguria) to 11.17 (Trento AP). The higher the odds, the higher the regional propensity of referring patients to a second-level vestibular test after a first-level assessment. The same table also provides the regional number and prevalence × 100 K population of consultant medical audiologists and audiology technicians registered in Italy in 2025, whose nationwide supply was 2.16 and 1.82 × 100 K population, respectively. The regional differences were wide, ranging from 0.51 × 100 K in Sardinia to 3.73 × 100 K in Veneto for consultant medical audiologists, and from 0.66 × 100 K in Liguria to 2.76 × 100 K Emilia Romagna for audiology technicians.

**Figure 6:**
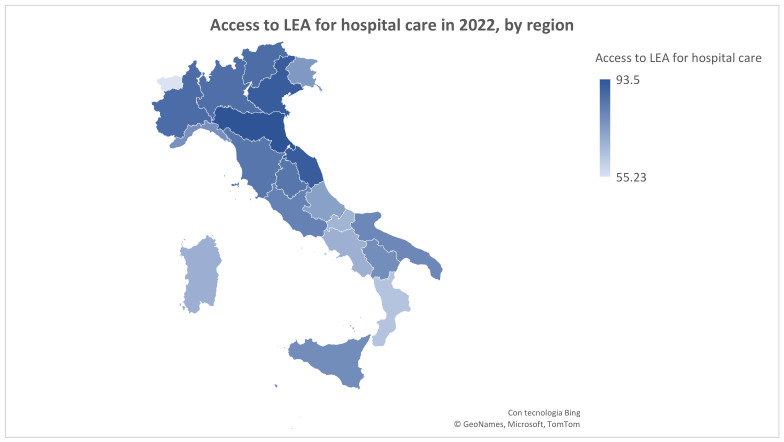
Access to LEA for hospital care in 2022, by Italian region.

**Figure 7:**
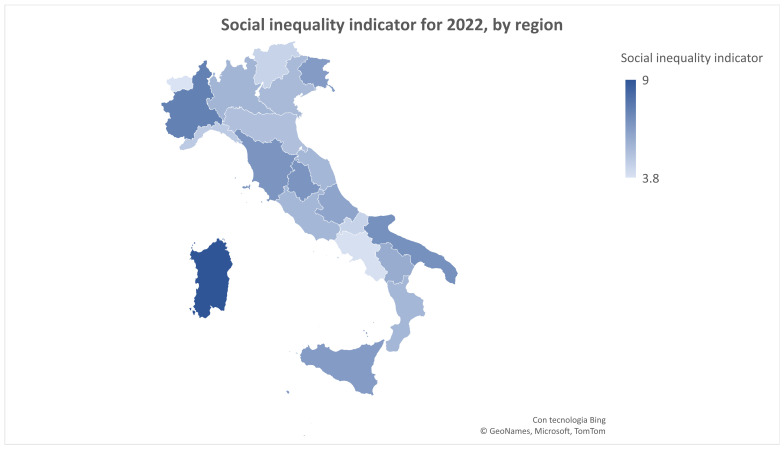
Social inequality indicator for year 2022, by Italian region.

**Figure 8:**
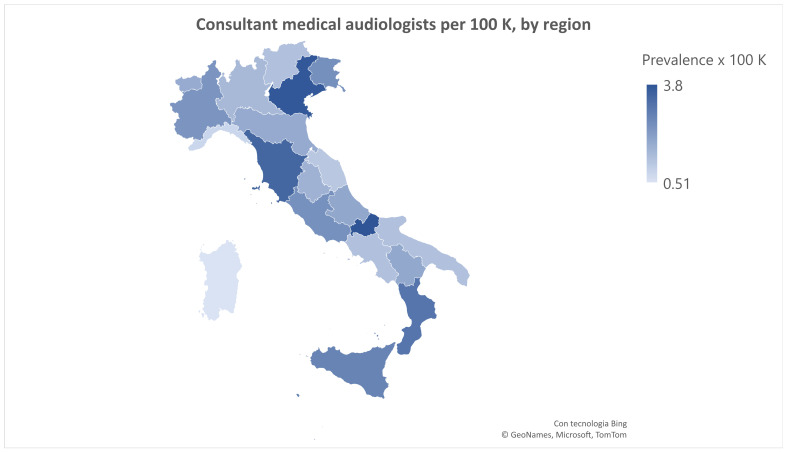
Prevalence of consultant medical audiologists registered in Italy in 2025 per 100 k population, by Italian region.

**Figure 9:**
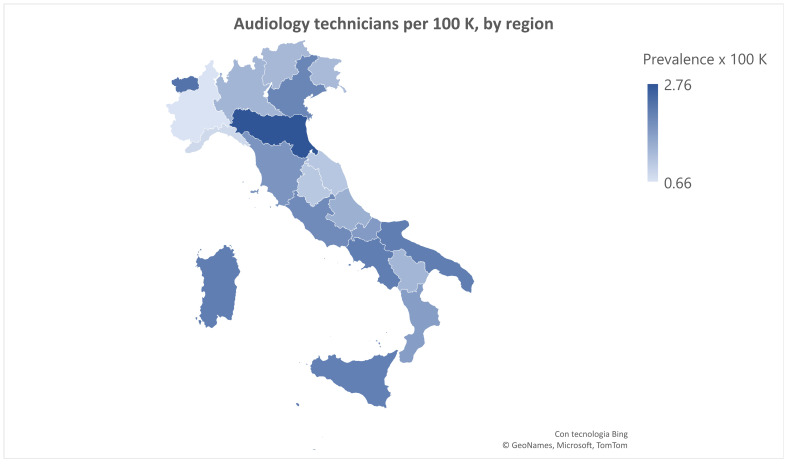
Prevalence of audiology technicians registered in Italy in 2025 per 100 k population, by Italian region.

Table 4 also shows the regional indicators of LEA in three healthcare domains (prevention care, district healthcare, and hospital care) and the social inequality indicator for calendar year 2022. The distribution was uneven. The score of access to LEA for prevention care ranged from 36.59 (Calabria) to 94.27 (Province of Trento); LEA for district healthcare varied from 34.88 (Calabria) to 96.40 (Veneto); LEA for hospital care ranged from 55.23 (Aosta Valley) to 98.35 (Province of Trento); the social inequality indicator varied from 3.60 (Province of Bolzano) to 9.00 (Sardinia). The regional geographical distribution of the latter social inequality indicator, the indicator of access to LEA for hospital care, and prevalence × 100 K population of consultant medical audiologists and technicians can be visualized in Figure 6, Figure 7, Figure 8 and Figure 9.

Table 5 displays the output of linear regression analysis on the prevalence of second-level vestibular tests × 100 K population. For consistency, the six regression models of all three study years included the same predictors, backward stepwise selected for 2021 and 2022, although the procedure retained only LEA for hospital care in 2023.

The results are shown in two columns (PAF and total facilities funded by the Italian NHS), three groups of rows (years 2021, 2022 and 2023), and three predictors at each row and column interception (social inequality indicator for 2022; access to LEA for district healthcare in 2022; access to LEA for hospital care in 2022). The model corresponding to PFPAF yielded no significant results in any of the three study years. Results of both columns in any of the three study years suggested consistent associations between the prevalence of second-level vestibular tests × 100 K population and the social inequality indicator (largely negative effect size), LEA for hospital care (positive effect and strong significance) and LEA for district healthcare (negative effect, although not significant for year 2023).

The F-test was consistently significant in all six multiple regression models, despite the low number of complete case analysis observations.

Notice the lack of an analogue table on the regional prevalence of first-level vestibular tests, since the corresponding results of regression analysis were never significant.

Table 6 displays the results of a multivariable logistic regression model. All other Italian regions but Veneto (aOR = 4.803; 95%CI: 4.424–5.214) and Trento AP (aOR = 1.437; 1.362–1.622) were less likely than Lombardy (reference) to refer patients to any of the three second-level examinations (recorded spontaneous nystagmus, induced nystagmus or rotary stimulation tests) following a first-level vestibular assessment. Aosta Valley was excluded by default from the latter model, since it did not perform any second-level vestibular test in any of the 3 study years. All regional differences in referral propensity were highly significant. Adjusting for region, referral propensity progressively reduced in 2022 (aOR = 0.893; 0.869–0.918) and 2023 (aOR = 0.828; 0.805–0.851) with respect to 2021.

## 4. Discussion

### 4.1. Regional and Yearly Differences

Defining the geographic variation in healthcare utilization is a relevant mission of clinical epidemiology [31], allowing one to identify potential barriers leading to the underutilization or overutilization of services, useful for establishing priorities for audits, healthcare planning, and resource allocation. Theoretically, the healthcare supplied to patients should be consistent across different regions. However, differences in population characteristics may explain any variation in healthcare delivered [32,33].

Prevalence of first-level vestibular examinations × 100 K increased from 121.08 in 2021 to 128.47 in 2022 and 129.02 in 2023. As with audiometry tests, it can be reasonably argued that these increases reflected the recovery of healthcare services following the COVID-19 disruption [34]. However, prevalence of second-level vestibular tests × 100 K was marginally reduced from 38.24 in 2021 to 37.62 in 2022 and 37.02 in 2023. The latter figure is the result of a progressively higher supply over time of second-level tests delivered in PAF accompanied by their declined delivery in PFPAF. Whilst increasing over time in PAF (from 21.19 in 2021 to 23.82 in 2022 and 24.31 in 2023), the nationwide prevalence × 100 K population of second-level vestibular examinations was in fact slightly reduced in PFPAF (from 17.06 in 2021 to 13.80 in 2022 and 12.72 in 2023) (Table 1, Table 2 and Table 3). This again may reflect post-pandemic adjustments, with shifts in public funding and improved access to audiology services.

Considerable regional variation in the supply of both first- and second-level vestibular assessments was observed in the present study. Strong regional differences in LEA delivery are already an established issue in Italy, with territorial discrepancies not only between the north and south of the country but also among regions in the north-east, north-west, and center [28].

The total nationwide number of audiology tests performed during 2021–2023 was 223,250 (77%) and 66,600 (23%), for first- and second-level vestibular testing, respectively, with significant regional variation. Presuming vestibular function assessment as a sequential process, a first potential problem could be the lack of diagnosis, i.e., insufficient instrumental testing in a proportion of the general population owing to the underutilization of services or barriers in accessing audiology services within the Italian NHS.

An interview-based study conducted in China reported that most consultant medical audiologists were primarily engaged in audiometric assessments. Although most respondents had received proper clinical audiology training, with 98.77% holding a medical degree or higher education, their tasks closely resembled that of audiology technicians [35]. The situation in Italy could be similar, since both countries have systems providing universal healthcare, explaining why the above shortfall was more relevant for instrumental tests than first-level vestibular examinations. The former tests are often performed in specialized hospitals or audiology centers, using equipment like special goggles and rotary chairs to check the inner ear’s balance function, with the goal of diagnosing dizziness or balance disorders [35]. In a study conducted in three Italian municipalities of Central Italy, the incidence of vestibular neuritis was significantly higher in patients living in urban compared to rural areas, probably due to easier access to oto-neurological referral centers [36].

### 4.2. Referral Propensity to Instrumental Vestibular Tests

Prevalence of second-level vestibular examinations as well as the regional propensity of referring patients to a second-level vestibular test after a first-level assessment were considerably uneven across the various Italian regions, probably reflecting discrepancies in access to hospital care.

Instrumental (second-level) vestibular tests are more costly than first-level clinical evaluations of the vestibular function. This likely explains why the regional prevalence of first-level vestibular assessments was not influenced by any indicator of social inequality or access to healthcare. By contrast, significant associations were found between referral propensity to second-level vestibular tests following a first-level vestibular assessment and three social determinants of access to healthcare. Increasing social inequality reflects a higher share of the population reporting barriers in accessing healthcare services within the Italian NHS and hinges on socio-economic status; as a result, the association between the social inequality indicator and prevalence of second-level vestibular tests was largely negative. By contrast, an increasing score of LEA for hospital care reflects higher access to this type of healthcare; as a result, the respective association with the regional prevalence of second-level vestibular tests was positive. An increasing indicator of access to district healthcare reflects higher access to primary care services; as a result, the association with the regional prevalence of second-level vestibular tests was negative.

Moreover, in logistic regression, the odds ratio of referral propensity to a second-level vestibular examination progressively decreased from 2021 to 2023 and was significantly higher in three northern regions, Lombardy, Trento AP and Veneto, paradoxically the region with the lowest supply of first-level vestibular examinations after Aosta Valley in any of the three study years. Of note, Lombardy exhibited by far the highest prevalence of second-level examinations delivered in PFPAF.

The lack of confirmatory data on the prevalence of vestibular examinations in large cohorts led us to seek a comparison with incidence data from the general population.

Only few and recent studies analyzed the incidence of peripheral vestibular disorders (PVDs). To the best of our knowledge, the first study investigating the incidence of PVD was carried out in Taiwan. All patients aged ≥ 20 years who received a first-time diagnosis of PVD between 2010 and 2018 were identified from Taiwan’s Longitudinal Health Insurance Database. Annual incidence rates per 100 K population were obtained by dividing the annual incident PVD cases by the corresponding total population aged ≥ 20 years. Mean annual incidence rate × 100 K was as follows: 216.1 (min: 124.4 to max: 346.7) for Meniere’s disease (MD); 198.5 (min: 169.5 to max: 233.5) for benign paroxysmal positional vertigo (BPPV); 136.4 (min: 90.7 to max: 208.2) for vestibular neuritis (VN); 938.6 (min: 658.7 to max: 1193.4) for other or unspecified peripheral vestibular dizziness; 1489.6 (min: 1043.6 to max: 1981.7). Females exhibited consistently higher mean annual incidence than males for all types of PVDs. In both sexes, the incidence decreased substantially year by year during 2010–2018 [37].

Another study from South Korea used National Health Insurance Service data to investigate the yearly incident cases of PVD, such as BPPV, VN, and MD [38]. Patients undergoing canalolith repositioning maneuver under BPPV diagnosis were defined as patients with BPPV. Patients undergoing vestibular function tests once or more at admission or re-assessed once or more in outpatients within one month under VN diagnosis were defined as diagnosed with VN. Patients undergoing both audiometric and vestibular function tests once or more at admission or re-assessed once or more within one month under MD diagnosis were defined as patients with MD. The number of incident cases of PVD in 2020 was as follows: 179,572 for BPPV; 32,611 for VN; and 26,199 for MD [38]. In view of the above, we considered only VN and MD (58,810 = 32,611 + 26,199) patients tested in South Korea in the year 2020. Considering the census population of South Korea registered in 2020 (N = 51,845,787), the prevalence of second-level vestibular examinations delivered for MD or VN in 2020 was 113.4 × 100 K population. On the other hand, the total number of second-level vestibular tests performed in Italy during 2021–2023 was 66,600 (Table 1, Table 2 and Table 3), for an average yearly number of 22,200 (=66,600/3) tests, which were assumed to be performed only once to establish a diagnosis of either VN or MD. Considering the Italian census population on 31 December 2023 (N = 58,971,230), 66,873 (=(58,971,230/100,000) × 113.4)) second-level vestibular examinations would be needed to match the same prevalence (113.4 × 100 K) of tests supplied in South Korea in 2020. Out of 66,873, only 22,200 tests were actually delivered in Italy. Therefore, the difference (66,873 − 22,200 = 44,673) corresponds to the number of patients who may have given up vestibular evaluation or turned to private audiological services with out-of-pocket payments in Italy. This quota equaled 66.8% (=44,673/66,873), assuming an incidence of peripheral vestibular disorders matching that of the general population of South Korea. The latter percentage is likely higher than 66.8%, considering re-evaluation of vestibular tests within one month required for the diagnosis of VN and MD, and the vestibular tests (such as nystagmus) sometimes necessary to confirm a BPPV diagnosis. Therefore, the number of vestibular diagnostic tests supplied by the NHS (PAF and PFPAF) was probably severely insufficient to meet the needs of the audiology care of the general population, thereby likely forcing more than 67% of patients to give up vestibular evaluation or turn to private audiology services with out-of-pocket payment. However, as mentioned above, the Italian NHS is funded by central taxation, whereas the South Korean health system relies on mandatory social insurance. This fundamental difference inevitably influences the supply of healthcare within both latter universal health systems.

Furthermore, while regional testing shortfalls suggest unmet needs, higher utilization in hospital-dense regions may partly reflect supply-driven care, and lower referral rates in areas with stronger district-level services could reflect efficient primary care management rather than under-provision.

A hypothesis of missing diagnoses of vestibular disorders seems less likely than out-of-pocket payment. Indeed, according to recent analyses based on data from ISTAT, healthcare spending directly paid by Italian families (out-of-pocket payment) reached a record EUR 46.4 billion in 2024, an estimated 7.7% increase compared to the previous year [39]. The increase in private spending, which brings the Italian per capita average to approximately EUR 1115 (higher than the OECD average of EUR 906), reflects a growing demand for specialized healthcare services. Indeed, over 47% of private spending is concentrated on specialist visits and outpatient diagnostics, areas where fully private healthcare facilities (accessible by out-of-pocket payment) and publicly funded private healthcare centers in Italy guarantee care excellence standards, contributing to reducing waiting times and easing the congestion of patient accesses to the Italian NHS [40]. Again, since the Italian NHS is funded by central taxation, healthcare spending directly paid by families shall not be justified.

A retrospective review of a population-based medical records linkage system for residents of Olmsted County (Minnesota, USA) reported 53 patients (34 women and 19 men; mean age 51 years) with BPPV diagnosed in 1984. The yearly age- and sex-adjusted incidence was 64 per 100 K population (95%CI: 46–81). The incidence increased by 38% with each decade of life (95%CI: 23–54%) [41]. The large difference between the latter American study and the above estimates from Taiwan and South Korea is probably due to differences in study period, age of patients and healthcare systems. The Taiwan and South Korean systems are in fact near-universal health single-payer systems with mandatory payment of social health insurance, whereas the US has a private and voluntary health system.

Prevalence of dizziness or imbalance was reportedly around 20% among individuals older than 65 during the 12 months preceding a survey conducted in the US [42]. Since about 15 million Italians are older than 65, approximately 3 million elderly people require audiological care to identify presbiastasis, a condition increasing the risk of falls by compromising walking ability. Before reaching the final diagnosis, patients with peripheral vestibular disorders may in fact go through multiple clinical consultations and costly examinations such as magnetic resonance imaging [43]. Vestibular vertigo and balance instability are reportedly among the main causes of avoidable sickness absence and direct healthcare costs in the US [44,45].

### 4.3. Prospects

In Italy, the National Recovery and Resilience Plan (PNRR)—approved by the Council of the European Union on 8 December 2023—allocated EUR 2 billion to create 1038 primary care country hospitals within 2026 by legislative decree n.29 of 15 March 2024 [28,46]. These primary care facilities, designed to offer social and healthcare services to promote active aging and domiciliary care for long-term conditions, could also play a critical role in the prevention and diagnosis of vestibular disorders among elderly patients, through a multi-disciplinary integrated approach involving various specialists (audiologists, geriatricians, neurologists, physiotherapists, etc.), thereby improving the efficiency of healthcare.

A recent study from Johns Hopkins Hospital, using a smartphone-based eye tracking system (Tele-Dizzy Eye-Phone), enabled early detection of positional nystagmus with vertigo, with a positive predictive value of 100% (95%CI = 43–100%) and a negative predictive value of 100% (95%CI = 84–100%) [47]. A simple registration of ocular movements by artificial intelligence on smartphones, submitted to a cloud platform, allows one to analyze the low phase speed of ocular movements, identifying the intensity, duration, and direction of any nystagmus remotely during virtual examinations. In pilot studies, the diagnostic accuracy of artificial intelligence was comparable to video-nystagmography (VNG) [48,49]. Telemedicine may, therefore, represent an effective, promising, and cost-effective tool for detecting positional nystagmus remotely, offering prospects to increase access to audiology services, particularly for residents of rural and peripheral areas of Italy.

## 5. Strengths and Limitations

### 5.1. Strengths

This is a nationwide study, providing evidence on the distribution of vestibular tests across the various Italian regions during 2021–2023. The findings of this study can be used to guide health policies addressing the performance of the Italian NHS. The topic covered by this study is largely neglected in the open literature.

The relatively small number of observational units, corresponding to the 21 Italian regions and APs, combined with multiple predictors increases the risk of overfitting and instability of estimates. However, the number of irrelevant or noisy (independent) variables was reduced through stepwise selection, and if all variables displayed in Table 2 were retained in the three linear regression models, significant terms were still the social inequality indicator, level of access to hospital healthcare and level of access to district healthcare.

Moreover, the results of liner regression analysis were consistent by calendar year (2021–2023) and type of audiology facilities. Furthermore, the sign of the regression coefficients was consistently positive for “LEA for hospital care 2022” (the main predictor throughout) and consistently negative for “Social inequality indicator year 2022” and “LEA for district health care 2022”. Finally, while standard tests are conducted at the 0.05 significance level, “LEA for hospital care 2022” would remain a significant predictor even by strengthening the significance level from 0.05 to 0.009 in four out of six regression models.

### 5.2. Limitations

A relevant limitation of the present study is the ecological design, which could have constrained the inference and causality of association due to ecological fallacy.

The referral propensity to a second-level instrumental test was a proxy indicator, relying on the assumption that all second-level tests are preceded by a first-level assessment, which may not systematically hold, since a general practitioner or a consultant medical may in some cases even refer a patient directly for an instrumental vestibular test, by-passing a first-level assessment.

In fact, the ecological study design prevented us from accessing information on the timeline of vestibular assessments, i.e., whether a first-level assessment preceded a second-level test. However, it is, overall, sensible to assume that a patient undergoes an instrumental vestibular examination following a first-level evaluation, especially since this study only considered audiology care supplied in PAF and PFPAF. In the Italian NHS, without referral by a primary care physician or another medical consultant direct, specialist access is possible only in fully private healthcare centers, with out-of-pocket payment. Therefore, the rationale for this referral propensity indicator is upheld. Moreover, the results on predictors of referral propensity in linear regression analysis were plausible.

Moreover, the propensity referral proxy cannot distinguish between repeat testing and new referrals and may be highly sensitive to small denominators.

While regional testing shortfalls suggest unmet need, higher utilization in hospital-dense regions may partly reflect supply-driven care, and lower referral rates in areas with stronger district-level services could reflect efficient primary care management rather than under-provision. Moreover, it should also be acknowledged that some patients may have not appropriately required further testing following a first-level vestibular assessment.

The timeline of indicators used in linear regression analysis was mismatched, since the prevalence of second-level vestibular tests referred to the year 2023, indicators of access to healthcare and social inequality referred to 2022 and prevalence of registered audiology professionals referred to 2025. However, we believe the impact of this temporal mismatch on the results is marginal.

The number of patients forgoing vestibular assessment was estimated based on the assumption of similar incidence of vestibular disorders between Italy and South Korea, another high-income country featuring universal healthcare and a similar population size. However, as already mentioned, the health systems of the latter two countries differ in their financial support, and the Italian and South Korean general populations may have different genetic predispositions to vestibular disorders.

Finally, unmeasured regional confounders, including urban–rural access gradients, specialist waiting times, private-sector spillover, underlying variation in prevalence of vestibular disease and co-morbidities, including low levels of vitamin D [50], may have influenced the observed associations. Given the ecological design, the direction and magnitude of such bias cannot be quantified, reinforcing the hypothesis-generating nature of these findings.

## 6. Conclusions

The number of vestibular function tests supplied within the Italian NHS during 2021–2023 was uneven across the various regions and probably severely insufficient to meet the audiological care needs of the general population. The shortfall was more relevant for more costly instrumental vestibular tests than for clinical evaluation of the vestibular function. Assuming an incidence of vestibular disorders similar to South Korea, another high-income country with a similar population size and universal healthcare, more than 67% of Italian patients affected by vestibular disorders may have indeed been forced to give up second-level vestibular tests or turn to fully private audiology services with out-of-pocket payment. However, some patients may not have required further testing following a first-level vestibular assessment. Moreover, while regional testing shortfalls suggested unmet needs, higher utilization in hospital-dense regions may partly reflect supply-driven care, and lower referral rates in areas with stronger district-level services could reflect efficient primary care management rather than under-provision. Expanding the diagnostic capacity should, therefore, be paired with optimized referral pathways and alignment with clinical evaluation to avoid low-value testing or diagnostic cascades.

The prevalence of second-level vestibular examinations as well as the regional propensity of referring patients to a second-level vestibular test after a first-level assessment were considerably uneven across the various Italian regions, probably reflecting discrepancies in access to hospital care.

Massive resources—supplied by the PNRR, funded by Next Generation EU funds— have been allocated to the Italian NHS as a major response to the COVID-19 pandemic. In particular, EUR 20.23 billion was allocated to Mission 6, with the aim of addressing services in relation to healthcare in every area of the country, improving infrastructure and technological endowments, promoting research and innovation, and developing the technical, digital, and managerial skills of staff. The Italian Government allocated an additional EUR 3.7 billion in 2020 and EUR 1.7 billion in 2021 to the NHS, increasing health expenditure by 3.3 percent and 1.7 percent, respectively, over the original funding plan [46].

Telemedicine may offer an effective and cost-effective tool for detecting positional nystagmus remotely, increasing access to audiology services, particularly for residents in rural and peripheral areas of Italy.

## Figures and Tables

**Table 1:** Distribution of vestibular tests (number and prevalence × 100 K population) performed in 2021, by region, level of examination, and type of facility. First level = vestibular clinical examination; second level = instrumental vestibular tests (recorded spontaneous nystagmus, vestibular caloric test or vestibular rotatory test). PAF = public audiology facilities; PFPAF = publicly funded private audiology facilities; AP = autonomous province; N = Number. Green highlights reflect regional prevalence > 100 × 100 K for first-level assessments (darker green for prevalence > 150 × 100 K) and >50 × 100 K for second-level vestibular tests (darker green for prevalence > 100 × 100 K).

Region	1st Level Vestibular Examinations (Year 2021)						2nd Level Vestibular Examinations (Year 2021)					
	PAF		PFPAF		Total		PAF		PFPAF		Total	
	N	×100 K	N	×100 K	N	×100 K	N	×100 K	N	×100 K	N	×100 K
**Piedmont**	3340	78.61	548	12.87	3888	91.45	269	6.33	80	1.88	349	8.21
**Aosta Valley**	5	4.06	0	0.00	5	4.07	0	0	0	0	0	0
**Lombardy**	8037	80.56	3935	39.58	11,972	119.58	3528	35.36	5391	54.04	8919	89.40
**Liguria**	2536	168.21	0	0.00	2536	168.04	19	1.26	0	0	19	1.26
**Bolzano AP**	871	163.06	42	7.89	913	169.85	346	64.78	52	9.74	398	74.51
**Trento AP**	848	156.17	160	29.58	1008	184.90	756	139.23	0	0	756	139.23
**Veneto**	1942	40.04	141	2.91	2083	42.93	1673	34.50	179	3.69	1852	38.19
**FVG**	963	80.64	65	5.44	1028	86.05	241	20.18	0	0	241	20.18
**Emilia-Romagna**	3825	86.20	71	1.60	3896	87.51	2390	53.86	0	0	2390	53.86
**Tuscany**	7958	217.31	110	3.00	8068	220.41	597	16.30	0	0	597	16.30
**Umbria**	772	90.14	0	0.00	772	90.50	6	0.70	0	0	6	0.70
**Marche**	1264	85.16	63	4.24	1327	89.50	164	11.05	0	0	164	11.05
**Lazio**	4669	81.62	4021	70.36	8690	152.06	509	8.90	404	7.06	913	15.96
**Abruzzo**	1212	95.24	104	8.15	1316	103.66	203	15.95	48	3.77	251	19.72
**Molise**	678	233.28	44	15.06	722	249.63	81	27.87	0	0	81	27.87
**Campania**	4585	81.74	1246	22.15	5831	103.66	419	7.47	589	10.50	1008	17.97
**Apulia**	2894	74.06	22	0.56	2916	74.95	34	0.87	2	0.05	36	0.92
**Basilicata**	641	119.24	77	14.23	718	134.65	13	2.42	19	3.53	32	5.95
**Calabria**	1391	75.33	410	22.20	1801	97.96	837	45.33	2	0.11	839	45.43
**Sicily**	3884	80.68	5587	115.59	9471	197.42	361	7.50	3303	68.61	3664	76.11
**Sardinia**	2331	147.70	110	6.93	2441	155.68	61	3.87	0	0	61	3.87
**ITALY**	**54,626**	**92.57**	**16,746**	**28.40**	**71,402**	**121.08**	**12,507**	**21.19**	**10,069**	**17.06**	**22,576**	**38.24**

**Table 2:** Distribution of vestibular tests (number and prevalence × 100 K population) performed in 2022, by region. First level = vestibular clinical examination; second level = instrumental vestibular tests (recorded spontaneous nystagmus, vestibular caloric test or vestibular rotatory test). PAF = public audiology facilities; PFPAF = publicly funded private audiology facilities; AP = autonomous province; N = Number. Green highlights reflect regional prevalence > 100 × 100 K for first-level assessments (darker green for prevalence > 150 × 100 K) and >50 × 100 K for second-level vestibular tests (darker green for prevalence > 100 × 100 K).

Region	1st Level Vestibular Examinations (Year 2022)						2nd Level Vestibular Examinations (Year 2022)					
	PAF		PFPAF		Total		PAF		PFPAF		Total	
	N	×100 K	N	×100 K	N	×100 K	N	×100 K	N	×100 K	N	×100 K
**Piedmont**	3258	76.54	395	9.28	3653	85.82	435	10.22	46	1.08	481	11.30
**Aosta Valley**	46	37.29	0	0	46	37.44	0	0	0	0	0	0
**Lombardy**	8725	87.75	3702	37.23	12,427	124.12	4184	42.08	4192	42.16	8376	84.24
**Liguria**	2796	185.26	0	0	2796	185.27	11	0.73	0	0	11	0.73
**Bolzano AP**	638	119.79	16	3.00	654	121.67	359	67.40	19	3.57	378	70.97
**Trento AP**	846	156.39	103	19.04	949	174.07	871	161.01	0	0	871	161.01
**Veneto**	2465	50.85	94	1.94	2559	52.74	2287	47.18	34	0.70	2321	47.88
**FVG**	1434	120.04	45	3.77	1479	123.81	318	26.62	0	0	318	26.62
**Emilia-Romagna**	3507	79.25	12	0.27	3519	79.04	2288	51.70	0	0	2288	51.70
**Tuscany**	7317	199.74	55	1.50	7372	201.39	614	16.76	0	0	614	16.76
**Umbria**	701	81.62	0	0	701	82.17	6	0.70	0	0	6	0.70
**Marche**	1388	93.33	68	4.57	1456	97.91	238	16.00	0	0	238	16.00
**Lazio**	6232	109.05	5396	94.42	11,628	203.64	481	8.42	640	11.20	1121	19.62
**Abruzzo**	1177	92.24	88	6.90	1265	99.64	199	15.60	99	7.76	298	23.36
**Molise**	523	179.02	21	7.19	544	188.09	15	5.13	0	0	15	5.13
**Campania**	6579	116.97	1051	18.69	7630	136.40	245	4.36	376	6.69	621	11.04
**Apulia**	3006	76.63	20	0.51	3026	77.78	183	4.66	1	0.03	184	4.69
**Basilicata**	673	124.36	54	9.98	727	136.34	10	1.85	31	5.73	41	7.58
**Calabria**	1479	79.71	481	25.92	1960	106.60	879	47.37	2	0.11	881	47.48
**Sicily**	4257	88.08	4774	98.77	9.031	188.25	337	6.97	2700	55.86	3037	62.83
**Sardinia**	2238	140.98	103	6.49	2341	149.47	92	5.80	0	0	92	5.80
**ITALY**	**59,285**	**100.49**	**16,478**	**27.93**	**75,763**	**128.47**	**14,052**	**23.82**	**8140**	**13.80**	**22,192**	**37.62**

**Table 3:** Distribution of audiology tests (number and prevalence × 100 K population) performed in 2023, by region. First level = vestibular clinical examination; second level = instrumental vestibular tests (recorded spontaneous nystagmus, vestibular caloric test or vestibular rotatory test). PAF = public audiology facilities; PFPAF = publicly funded private audiology facilities; AP = autonomous province; N = Number. Green highlights reflect regional prevalence > 100 × 100 K for first-level assessments (darker green for prevalence > 150 × 100 K) and >50 × 100 K for second-level vestibular tests (darker green for prevalence > 100 × 100 K).

Region	1st Level Vestibular Examinations (Year 2023)						2nd Level Vestibular Examinations (Year 2023)					
	PAF		PFPAF		Total		PAF		PFPAF		Total	
	N	×100 K	N	×100 K	N	×100 K	N	×100 K	N	×100 K	N	×100 K
**Piedmont**	3503	82.45	160	3.76	3663	86.16	573	13.48	54	1.27	627	14.75
**Aosta Valley**	3	2.44	0	0	3	2.44	0	0	0	0	0	0
**Lombardy**	8883	89.04	3554	35.74	12,437	124.22	4008	40.03	4247	42.42	8255	82.45
**Liguria**	3029	200.91	0	0	3029	200.71	14	0.93	0	0	14	0.93
**Bolzano AP**	672	125.81	41	7.70	713	132.64	493	91.72	40	7.44	533	99.16
**Trento AP**	950	174.96	115	21.26	1065	195.35	696	127.67	0	0	696	127.67
**Veneto**	3095	63.82	89	1.84	3184	65.62	2939	60.57	42	0.87	2981	61.44
**FVG**	1304	109.19	50	4.19	1354	113.34	238	19.92	0	0	238	19.92
**Emilia-Romagna**	3621	81.60	19	0.43	3640	81.76	2424	54.45	0	0	2424	54.45
**Tuscany**	7290	199.07	42	1.15	7332	200.30	554	15.13	0	0	554	15.13
**Umbria**	883	103.11	0	0	883	103.51	5	0.59	0	0	5	0.59
**Marche**	1474	99.31	33	2.22	1507	101.64	209	14.10	1	0.07	210	14.16
**Lazio**	6423	112.28	5666	99.14	12,089	211.54	356	6.23	729	12.76	1085	18.99
**Abruzzo**	1068	83.92	66	5.17	1134	89.32	202	15.91	62	4.88	264	20.79
**Molise**	625	215.05	17	5.82	642	221.97	7	2.42	0	0	7	2.42
**Campania**	6672	118.94	1020	18.14	7692	137.51	216	3.86	384	6.86	600	10.73
**Apulia**	2886	73.85	18	0.46	2904	74.64	318	8.17	1	0.03	319	8.20
**Basilicata**	394	73.29	38	7.02	432	81.02	0	0	20	3.75	20	3.75
**Calabria**	1982	107.33	14	0.75	1996	108.56	813	44.22	2	0.11	815	44.32
**Sicily**	4124	85.67	3877	80.21	8001	166.78	164	3.42	1917	39.96	2081	43.38
**Sardinia**	2306	146.12	79	4.98	2385	151.87	104	6.62	0	0	104	6.62
**ITALY**	61,187	103.65	14,898	25.26	76,085	129.02	14,333	**24.31**	7499	**12.72**	21,832	**37.02**

**Table 4:** Regional distribution of the odds of number of [2nd/(1st − 2nd)] vestibular tests performed in 2022; regional indicator of social inequality and access to essential levels of care (LEA) in three healthcare domains (prevention care, district healthcare, hospital care) for year 2022; regional number and prevalence × 100 K population of consultant medical audiologists and technicians registered in Italy in 2025; Italian census population as of 31 December 2025 (Italian Office for National Statistics). N = Number; AP = autonomous province; FVG = Friuli Venezia Giulia. Orange highlights reflect LEA access < 60.

Area	Region	Odds 2nd/(1st − 2nd) Level Vestibular Tests (Year 2022)	LEA Access Domain (Year 2022) * Benchmark ≥ 60			Forgone Access to Healthcare due to Social Inequality (Year 2022) *	Consultant Medical Audiologists (Year 2025)		Audiology Technicians (Year 2025)		Italian Census Population on (31 December 2023)		
			Prevention Care	District Care	Hospital Care		N	Prevalence ×100 K	N	Prevalence ×100 K	N	Residents Aged >65 Years (Prevalence ×100 K)	Female Prevalence ×100 K
**North-West**	**Piedmont**	0.109	88.79	86.55	87.07	7.40	77	1.81	98	2.31	4,251,623	25.26	51.14
	**Aosta Valley**	-	48.48	47.25	55.23	3.80	1	0.81	1	0.81	122,877	24.02	50.91
	**Lombardy**	2.068	90.18	94.66	86.09	5.50	148	1.48	134	1.34	10,012,054	22.30	50.79
	**Liguria**	0.004	61.41	86.81	77.49	4.70	35	2.32	10	0.66	1,509,140	27.58	51.67
**North-East**	**Bolzano AP**	1.370	54.14	77.03	75.23	3.60	7	1.30	7	1.30	537,533	19.40	50.40
	**Trento AP**	11.17	94.27	76.45	98.35	5.20	7	1.28	7	1.28	545,169	22.34	50.61
	**Veneto**	9.752	94.08	96.40	91.36	5.30	181	3.73	99	2.04	4,852,216	23.15	50.78
	**FVG**	0.274	71.24	73.30	75.29	6.40	21	2.43	15	1.26	1,194,616	25.83	51.13
	**Emilia-Romagna**	1.859	96.13	95.57	93.50	5.10	82	1.84	123	2.76	4,451,938	23.39	50.97
**Centre**	**Tuscany**	0.091	79.59	83.88	84.42	6.70	124	3.39	67	1.83	3,660,530	25.13	51.38
	**Umbria**	0.009	79.59	83.88	84.42	6.70	14	1.64	9	1.06	853,068	25.72	51.33
	**Marche**	0.195	60.91	91.03	91.26	5.40	17	1.15	16	1.08	1,482,746	24.88	51.04
	**Lazio**	0.110	74.08	72.07	81.30	5.40	138	2.41	112	1.96	5,714,745	22.12	51.52
**South**	**Abruzzo**	0.308	49.31	62.18	73.10	6.10	24	1.89	18	1.42	1,269,571	24.28	51.01
	**Molise**	0.028	50.69	61.23	67.54	4.40	11	3.80	5	1.73	289,224	25.39	50.45
	**Campania**	0.089	69.68	55.76	68.66	6.80	51	1.31	84	2.16	3,890,661	19.64	51.14
	**Apulia**	0.065	75.97	70.02	79.69	6.80	51	1.31	84	2.16	3,890,661	22.94	51.22
	**Basilicata**	0.060	68.46	61.92	78.03	5.80	10	1.88	7	1.32	533,233	23.98	50.57
	**Calabria**	0.816	36.59	34.88	63.78	5.40	56	3.05	31	1.69	1,838,568	22.65	50.98
**Islands**	**Sicily**	0.507	47.18	58.04	78.38	6.40	130	2.71	102	2.13	4,797,459	21.92	51.23
	**Sardinia**	0.041	46.55	50.45	69.11	9.00	8	0.51	34	2.16	1,570,453	25.35	50.89

* https://www.salute.gov.it/new/sites/default/files/imported/C_17_pubblicazioni_3456_allegato.pdf (last accessed on 3 May 2026).

**Table 5:** Multivariate linear regression on regional prevalence × 100 K population of second-level (instrumental) vestibular tests, by calendar year (2021–2023). Adjusted regression coefficients (aRC) with 95% confidence intervals (95%CI). PAF = public audiology facilities; LEA = essential level of care guaranteed by the Italian National Health Service.

Predictors	Total Audiology Facilities Funded by the Italian NHS		PAF	
	aRC (95%CI)	*p*-Value	aRC (95%CI)	*p*-Value
**YEAR 2021**	**F test *p* = 0.018; R^2^ = 0.438**		**F test *p* = 0.006; R^2^ = 0.514**	
Social inequality indicator (year 2022)	−12.92 (−24.66; −1.19)	0.033	−13.04 (−22.72; −3.36)	0.011
LEA for district healthcare (year 2022)	−1.78 (−3.28; −0.28)	0.023	−1.67 (−2.91; −0.43)	0.001
LEA for hospital care (year 2022)	3.92 (1.44; 6.41)	0.004	3.70 (1.65; 5.75)	0.011
**YEAR 2022**	**F test *p* = 0.007; R^2^ = 0.500**		**F test *p* = 0.006; R^2^ = 0.515**	
Social inequality indicator (year 2022)	−12.41 (−24.18; −0.65)	0.040	−12.83 (−23.86; −1.79)	0.025
LEA for district healthcare (year 2022)	−2.01 (−3.52; −0.51)	0.012	−1.87 (−3.28; −0.46)	0.013
LEA for hospital care (year 2022)	4.58 (2.09; 7.07)	0.001	4.33 (2.00; 6.67)	0.001
**YEAR 2023**	**F test *p* = 0.030; R^2^ = 0.402**		**F test *p* = 0.022; R^2^ = 0.425**	
Social inequality indicator (year 2022)	−12.11 (−23.95; −0.27)	0.046	−12.09 (−23.12; −1.06)	0.034
LEA for district healthcare (year) 2022	−1.27 (−2.79; −0.24)	0.095	−1.25 (−2.67; 0.16)	0.078
LEA for hospital care (year 2022)	3.37 (0.87; 5.88)	0.011	3.29 (0.95; 5.62)	0.009

**Table 6:** Logistic regression model on the referral propensity to a second level vestibular test following a first level assessment—odds of any second-level vestibular tests (recorded spontaneous nystagmus, induced nystagmus, rotary stimulations) out of the difference between number of first-and second-level vestibular tests, by region. Odds ratio (aOR) adjusted for calendar year and region, with 95% confidence interval (95%CI). AP = autonomous province. Green colour highlights regions with significantly higher referral propensity (OR > 1); darker green reflects OR > 2.

AREA	REGIONS	aOR (222,695 obs.)	(95%CI)
**North-West**	**Piedmont**	0.056	0.052; 0.059
	**Aosta Valley**	-	-
	**Lombardy**	*reference*	
	**Liguria**	0.002	0.002; 0.003
**North-East**	**Bolzano AP**	0.596	0.547; 0.650
	**Trento AP**	1.488	1.363; 1.624
	**Veneto**	4.826	4.445; 5.239
	**Friuli Venezia Giulia**	0.117	0.108; 0.127
	**Emilia Romagna**	0.801	0.766; 0.837
**Centre**	**Toscana**	0.037	0.035; 0.039
	**Umbria**	0.003	0.002; 0.005
	**Marche**	0.082	0.075; 0.090
	**Lazio**	0.048	0.046; 0.051
**South**	**Abruzzo**	0.128	0.118; 0.138
	**Molise**	0.025	0.021; 0.031
	**Campania**	0.053	0.050; 0.056
	**Apulia**	0.029	0.026; 0.032
	**Basilicata**	0.023	0.019; 0.028
	**Calabria**	0.352	0.033; 0.373
**Islands**	**Sicily**	0.219	0.212; 0.227
	**Sardinia**	0.017	0.015; 0.019
**Calendar** **year**	**2021**	*reference*	
	**2022**	0.893	0.869; 0.918
	**2023**	0.828	0.805; 0.851

## Data Availability

Some of the data analyzed in the current study are not publicly available due to privacy and ethical restrictions but can be requested from the National Agency for Regional Health Services (Agenas), the National Federation of Provincial Medical Councils (FNOMCeO) and the National Federation of the Council of Healthcare Professionals (FNOTSRM-PSTRP). Data from the Italian Office for National Statistics (Istat) and the Italian Ministry of Health analyzed are instead publicly available, as per citation provided in the manuscript.

## Supplementary Materials

The following supporting information can be downloaded at: https://www.mdpi.com/article/10.3390/healthcare14111544/s1, Table S1: Distribution of 2nd level audiology tests (spontaneous recorded nystagmus, vestibular caloric test, vestibular rotatory tests) by region, calendar year and facility (public vs. publicly funded private healthcare facilities). Numbers (N) and rates (×100,000). PFPAF = Privately funded private audiology facilities. AP = autonomous province. TAA = Trentino Alto Adige.
