# Supplementary material for: Balance Disorders: Insufficient Supply of Vestibular Examinations by the Italian National Health Service, 2021–2023
Source: Healthcare (Basel). 2026 Jun 1;14(11):1544. doi: 10.3390/healthcare14111544 (PMC13257187; doi:10.3390/healthcare14111544)
Supplement: Supplementary file 1 [file healthcare-14-01544-s001.zip › healthcare-4191925-supplementary.pdf]

Supplementary Table S1. Distribution of 2nd level audiology tests (spontaneous recorded nystagmus, vestibular caloric test, vestibular rotatory tests) by region, calendar year and facility (public vs publicly funded private healthcare facilities). Numbers (N) and rates (x 100.000). PAF= Public audiology facilities; PFPAF= Privately funded private audiology facilities. AP= autonomous province. TAA= Trentino Alto Adige.

AREA	REGION		Spontaneous nystagmus						Induced (caloric) nystagmus						Rotary chair stimulation					
			2021		2022		2023		2021		2022		2023		2021		2022		2023	
			PAF	PFPAF	PAF	PFPAF	PAF	PFPAF	PAF	PFPAF	PAF	PFPAF	PAF	PFPAF	PAF	PFPAF	PAF	PFPAF	PAF	PFPAF
North-West	Piedmont	N	58	80	123	46	89	54	44	0	103	0	104	0	167	0	209	0	380	0
		x 100 K	1.37	1.88	2.89	1.08	2.09	1.27	1.04	0.00	2.42	0.00	2.45	0.00	3.93	0.00	4.91	0.00	8.94	0.00
	Aosta Valley	N	0	0	0	0	0	0	0	0	0	0	0	0	0	0	0	0	0	0
		x 100 K	0.00	0.00	0.00	0.00	0.00	0.00	0.00	0.00	0.00	0.00	0.00	0.00	0.00	0.00	0.00	0.00	0.00	0.00
	Lombardy	N	1086	2013	1297	1799	1112	1811	1556	1752	1831	1507	1755	1510	886	1626	1056	886	1141	926
		x 100 K	10.89	20.18	13.04	18.09	11.11	18.09	15.60	17.56	18.41	15.16	17.53	15.08	8.88	16.30	10.62	8.91	11.40	9.25
	Liguria	N	6	0	7	0	4	0	9	0	1	0	4	0	4	0	3	0	6	0
		x 100 K	0.40	0.00	0.46	0.00	0.27	0.00	0.60	0.00	0.07	0.00	0.27	0.00	0.27	0.00	0.20	0.00	0.40	0.00
North-East	TAA	N	608	24	728	9	657	19	485	28	486	10	532	21	9	0	17	0	0	0
		x 100 K	56.45	2.23	67.81	0.84	60.68	1.75	45.03	2.60	45.27	0.93	49.14	1.94	0.84	0.00	1.58	0.00	0.00	0.00
	Bolzano AP	N	229	24	286	9	303	19	117	28	73	10	190	21	0	0	0	0	0	0
		x 100 K	42.87	4.49	53.70	1.69	56.37	3.53	21.90	5.24	13.71	1.88	35.35	3.91	0.00	0.00	0.00	0.00	0.00	0.00
	Trento AP	N	379	0	441	0	354	0	368	0	413	0	342	0	9	0	17	0	0	0
		x 100 K	69.80	0.00	81.52	0.00	64.93	0.00	67.77	0.00	76.35	0.00	62.73	0.00	1.66	0.00	3.14	0.00	0.00	0.00
	Veneto	N	449	73	583	0	865	0	970	106	1220	34	1392	42	254	0	484	0	682	0
		x 100 K	9.26	1.51	12.03	0.00	17.83	0.00	20.00	2.19	25.17	0.70	28.69	0.87	5.24	0.00	9.98	0.00	14.06	0.00
	FVG	N	241	0	318	0	238	0	0	0	0	0	0	0	0	0	0	0	0	0
		x 100K	20.18	0.00	26.62	0.00	19.92	0.00	0.00	0.00	0.00	0.00	0.00	0.00	0.00	0.00	0.00	0.00	0.00	0.00
	Emilia Romagna	N	769	0	744	0	693	0	1.351	0	1282	0	1490	0	270	0	262	0	241	0
		x 100 K	17.33	0.00	16.81	0.00	15.57	0.00	30.44	0.00	28.97	0.00	33.47	0.00	6.08	0.00	5.92	0.00	5.41	0.00
Center	Tuscany	N	404	0	396	0	337	0	0	0	0	0	0	0	193	0	218	0	217	0
		x 100 K	11.03	0.00	10.81	0.00	9.21	0.00	0.00	0.00	0.00	0.00	0.00	0.00	5.27	0.00	5.95	0.00	5.93	0.00
	Umbria	N	0	0	0	0	0	0	0	0	0	0	0	0	6	0	6	0	5	0
		x 100 K	0	0	0	0	0	0	0	0	0	0	0	0	0.70	0	0.70	0	0.59	0
	Marche	N	1	0	3	0	3	0	156	0	232	0	204	0	7	0	3	0	2	1
		x 100 K	0.07	0.00	0.20	0.00	0.20	0.00	10.51	0.00	15.60	0.00	13.76	0.00	0.47	0.00	0.20	0.00	0.13	0.07
	Lazio	N	64	161	29	284	34	374	195	238	167	350	63	349	250	5	285	6	259	6
		x 100 K	1.12	2.81	0.51	4.97	0.59	6.54	3.41	4.16	2.92	6.12	1.10	6.11	4.37	0.09	4.99	0.10	4.53	0.10

South	Abruzzo	N	1	14	0	48	0	45	202	5	199	30	202	5	0	29	0	21	0	12
		x 100 K	0.08	1.10	0.00	3.76	0.00	3.54	15.87	0.39	15.60	2.35	15.91	0.39	0.00	2.28	0.00	1.65	0.00	0.95
	Molise	N	28	0	7	0	2	0	48	0	7	0	4	0	5	0	1	0	1	0
		x 100 K	9.63	0.00	2.40	0.00	0.69	0.00	16.52	0.00	2.40	0.00	1.38	0.00	1.72	0.00	0.34	0.00	0.35	0.00
	Campania	N	89	231	62	159	111	158	93	214	60	144	33	150	237	144	123	73	72	76
		x 100 K	1.59	4.12	1.10	2.83	1.98	2.82	1.66	3.81	1.07	2.56	0.59	2.68	4.22	2.57	2.19	1.30	1.29	1.36
	Apulia	N	13	0	106	0	194	0	1	0	47	0	98	0	20	2	30	1	26	1
		x 100 K	0.33	0.00	2.70	0.00	4.99	0.00	0.03	0.00	1.20	0.00	2.52	0.00	0.51	0.05	0.76	0.03	0.67	0.03
	Basilicata	N	12	12	10	17	0	13	1	7	0	13	0	7	0	0	0	1	0	0
		x 100 K	2.23	2.23	1.85	3.14	0.00	2.44	0.19	1.30	0.00	2.40	0.00	1.31	0.00	0.00	0.00	0.18	0.00	0.00
	Calabria	N	426	0	436	0	401	0	396	0	422	0	389	0	15	2	21	2	23	2
		x 100 K	23.07	0.00	23.50	0.00	21.81	0.00	21.44	0.00	22.74	0.00	21.16	0.00	0.81	0.11	1.13	0.11	1.25	0.11
Islands	Sicily	N	169	1.657	156	1.422	83	962	140	1500	152	1232	64	847	52	146	29	46	17	108
		x 100 K	3.51	34.42	3.23	29.42	1.73	20.05	2.91	31.16	3.14	25.49	1.33	17.66	1.08	3.03	0.60	0.95	0.35	2.25
	Sardinia	N	26	0	31	0	15	0	35	0	60	0	73	0	0	0	1	0	16	0
		x 100 K	1.65	0.00	1.95	0.00	0.96	0.00	2.22	0.00	3.78	0.00	4.65	0.00	0.00	0.00	0.06	0.00	1.02	0.00
ITALY		N	5058	4289	5763	3793	5495	3455	6167	3878	6755	3330	6939	2952	2384	1954	2765	1036	3088	1132
		x 100 K	8.57	7.27	9.77	6.43	9.32	5.86	10.45	6.57	11.45	5.64	11.77	5.01	4.04	3.31	4.69	1.76	5.24	1.92
Grand Total (Italy)		N	**9347**		**9556**		**8950**		**10,045**		**10,085**		**9891**		**4338**		**3801**		**4220**	
		x 100 K	**15.83**		**16.20**		**1.52**		**17.02**		**17.09**		**16.77**		**16.76**		**7.35**		**6.45**	
